# Association of Pyridoxal 5′-Phosphate with Sleep-Related Problems in a General Population

**DOI:** 10.3390/nu14173516

**Published:** 2022-08-26

**Authors:** Lin Ge, Jia Luo, Liming Zhang, Xiao Kang, Dongfeng Zhang

**Affiliations:** Department of Epidemiology and Health Statistics, The School of Public Health of Qingdao University, No. 308 Ningxia Road, Qingdao 266071, China

**Keywords:** sleep quality, sleep duration, pyridoxal 5′-phosphate, vitamin B6, dose–response relationship, cross-sectional study

## Abstract

The evidence on the relationship of pyridoxal 5′-phosphate (PLP) with sleep-related problems is limited and controversial. Notably, there is a lack of studies on the general population and studies of the dose–response relationship. Therefore, we conducted a cross-sectional study to examine the associations between serum PLP concentration and sleep-related problems (sleep quality and sleep duration) in adults, using the data of the National Health and Nutrition Examination Survey 2005–2010. High-performance liquid chromatography (HPLC) was used to test PLP in blood samples. Sleep quality and sleep duration were based on self-reported data, with sleep quality categorized as sleep disorder, trouble falling asleep, waking up during the night, and daytime sleepiness. The primary analyses utilized logistic regression models and restricted cubic spline. Compared with the first quartile (Q1), the odds ratios (ORs) and 95% confidence intervals (CIs) of daytime sleepiness for the Q2 and Q3 of serum PLP concentrations were 0.76 (0.59–0.99) and 0.78 (0.62–0.98), respectively. The relationship was only significant for males. Furthermore, a non-linear dose–response relationship was observed between serum PLP concentration and the risk of daytime sleepiness. Compared with the normal sleep duration group, serum PLP concentrations were negatively associated with the risks of very short, short, and long sleep duration, with relative risk ratios (RRRs) of 0.58 (0.43–0.81) (Q4), 0.71 (0.61–0.83) (Q4) and 0.62 (0.34–0.94) (Q3), respectively. The average serum PLP concentrations were higher in people with normal sleep duration, suggesting a non-linear dose–response relationship. Our study indicated that serum PLP concentrations were negatively associated with daytime sleepiness, and this association may only exist in males. Moreover, it was also inversely related to abnormal sleep duration (very short, short, long) compared to normal sleep duration.

## 1. Introduction

Healthy sleep is essential for maintaining mental and physical functions [1,2,3,4,5]. However, millions of people around the world suffer from sleep-related problems, especially with the COVID-19 pandemic bringing many new challenges to sleep problems [6,7]. Inappropriate sleep duration and sleep disorders have been linked to numerous negative effects on the body, including digestive system problems [8], cardiovascular disease [9,10], diabetes [11], cancer [12], depression symptoms [13], and neurodegenerative diseases, such as Parkinson’s and Alzheimer’s [14,15]. Currently, factors that influence sleep are being explored, including genetics [16], environment [17], hormones [18], and various diseases [19,20,21]. However, much attention has been drawn to dietary factors that are self-selectable and modifiable. According to relevant reviews and studies, overall diet quality and some nutrients are associated with sleep [22,23,24,25,26].

Vitamin B6, mainly including pyridoxine, pyridoxal, and pyridoxamine, is a water-soluble vitamin with a wide range of sources. Notably, its antioxidant properties have attracted extensive attention [27,28]. Vitamin B6 deficiency can lead to dermatitis [29], neuropsychiatric symptoms [30], impaired immune function [31], and epilepsy [32,33]. The most widely used direct biomarker of vitamin B6 is pyridoxal 5′-phosphate (PLP), which is more reliable and representative [34]. As a coenzyme, PLP promotes the conversion of tryptophan to 5-hydroxytryptamine (5-HT) [35,36], which is considered to be a precursor of melatonin synthesis [37,38], suggesting that PLP may affect sleep.

Several studies have been conducted to explore the relationship between vitamin B6 and sleep. An observational study of older adults in Taiwan showed that people with poor sleep quality consumed less vitamin B6 than those with good sleep quality [39]. Another study suggested that vitamin B6, combined with other substances, was effective in treating mild to moderate insomnia [40]. However, a randomized controlled trial, using a vitamin B6-containing complex, found no significant difference in sleep quality between the complex and placebo [41]. Furthermore, an intervention study showed that vitamin B6 supplements did not significantly affect sleep in volunteers [42]. The participants of the above studies were mainly the elderly, patients, and volunteers, rather than the general population. Meanwhile, there are few studies on the relationship between serum PLP and sleep. More importantly, there was no clear dose–response relationship between serum PLP and sleep. Therefore, given these inconsistencies, differences, and unknown quantitative relationships, we conducted this cross-sectional study to examine the relationships between serum PLP concentrations and sleep-related problems, including sleep quality and sleep duration.

## 2. Materials and Methods

### 2.1. Participants

The National Health and Nutrition Examination Survey (NHANES) is a program of the National Center for Health Statistics to assess the health and nutritional status of the non-institutionalized civilian population in the United States. NHANES has been an ongoing survey since 1999. The survey conducts a sample survey of about 5000 nationally representative people every year, with a biennial release cycle. All participants provided informed consent. More information about NHANES can be found elsewhere [43].

**Figure 1:**
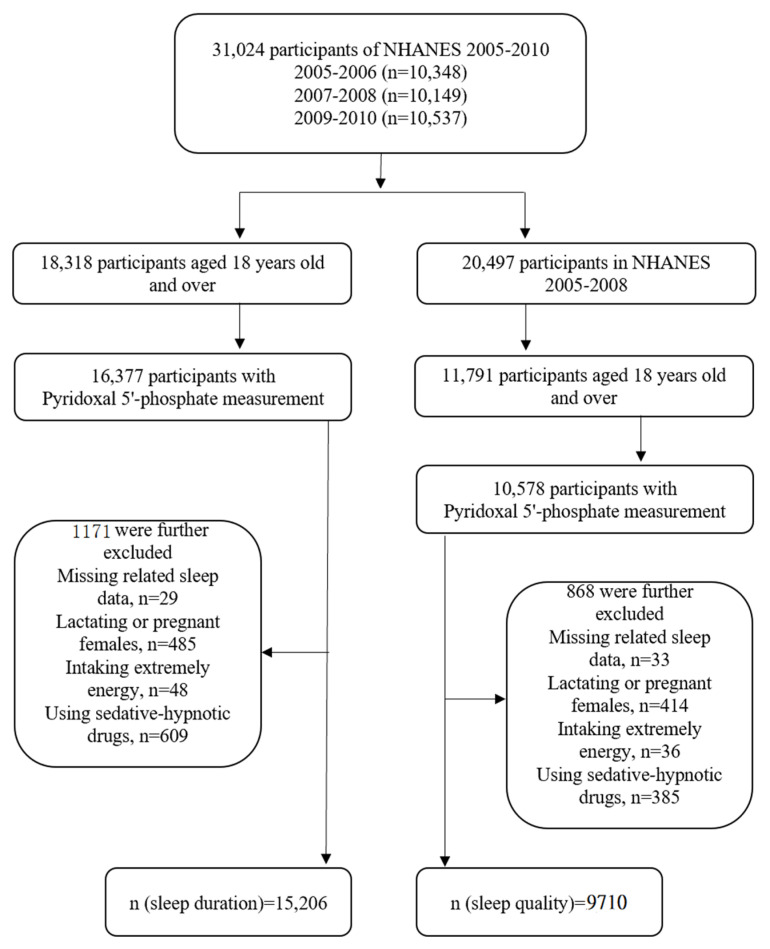
Flowchart of the screening process for the selection of eligible participants.

NHANES began to conduct sleep questionnaires in 2005–2006, and the sleep-related items were incomplete in 2009–2010. In addition, the measurement of serum PLP was up to 2019–2010, and the detection method was consistent between 2005 and 2010. Thus, in this study, two cycles of data (NHANES 2005–2006, and 2007–2008) and three cycles of data (NHANES 2005–2006, 2007–2008, and 2009–2010) were selected to explore sleep quality (*n* = 9710) and sleep duration (*n* = 15,206), respectively. We excluded participants <18 years old and those who did not have serum PLP data. Participants with extreme energy intake (500 kcal/day for both males and females, >8000 kcal/day for males, and >5000 kcal/day for females), females who were pregnant and lactating, and subjects using sedative-hypnotics were further excluded. Finally, 9710 people were included to study sleep quality and 15,206 were included to study sleep duration. The specific process is shown in Figure 1.

### 2.2. Sleep-Related Problems

We divided sleep-related problems into two dimensions: sleep quality and sleep duration. Sleep quality was categorized as sleep disorders, trouble falling asleep, waking up during the night, and daytime sleepiness in detail. The detailed definitions were as follows.

Sleep quality: Sleep disorders were defined as those diagnosed by a doctor or health professional [44]. Trouble falling asleep was defined as having trouble falling asleep more than five times in the past month—self-reported “often” or “almost always” (≥5 times a month) [45]. Wake up during the night was defined as self-reported “often” or “almost always” (≥5 times a month) waking up during the night and having trouble getting back to sleep in the past month [45]. Daytime sleepiness was defined as “often” or “almost always” (≥5 times a month) feeling excessively or overly sleepy (self-reported) during the day five or more times in the past month [46,47].

Sleep duration: The question was, “How much sleep do you usually get at night on weekdays or workdays?” According to the answers, sleep duration was classified into very short sleep (<5 h), short sleep (5–<7 h), normal sleep (7–<9 h), or long sleep (≥9 h) [26,48].

### 2.3. Serum Pyridoxal 5′-Phosphate Measurement

Blood samples were collected at the Mobile Examination Center (MEC). Serum PLP, which was the main active form and a reliable biomarker of vitamin B6 [34], was analyzed by high-performance liquid chromatography (HPLC) [49].

### 2.4. Covariates

Several demographic characteristics, lifestyles, dietary factors, and diseases were included as covariates to control potential confounding effects based on previous literature [18,26]: age, sex, races/ethnicities, the ratio of income to poverty, educational level, marital status, body mass index, physical activity, caffeine intake, energy intake, smoking status, drinking, hypertension, diabetes, depressive symptoms, and sampling seasons. Table S1 shows the detailed classification of covariates.

### 2.5. Statistical Analysis

To account for the complex sampling design of NHANES and make the results more representative, all statistical analyses were weighted in this study. Qualitative and non-normal quantitative data were described using numbers (weighted percentage) and median (quartile range), respectively. Participants were divided into quartiles based on their PLP levels. Chi-square test and Kruskal–Wallis test were used to test the difference among quartiles of PLP levels.

Serum PLP concentrations were divided into quartiles, with quartile 1 (Q1) as the reference. Binary logistic regression and multinomial logistic regression were used to assess the relationship between serum PLP and sleep-related problems, along with calculating the ES (effect size, OR, and RRR) and 95% confidence intervals (CIs). Only sex and age were adjusted in Model 1. Model 2 further adjusted for races/ethnicities, the ratio of income to poverty, educational level, marital status, body mass index, physical activity, caffeine intake, energy intake, smoking status, drinking, hypertension, diabetes, depressive symptoms, and sampling seasons. In the analysis of sleep duration by multinomial logistic regression, normal sleep duration (7–<9 h) was used as the reference. In addition, taking into account sex and age differences in sleep [50,51], we performed stratified analyses based on these two factors. Finally, the dose–response relationship between serum PLP concentration and risk of sleep quality problems was explored using restricted cubic spline with three knots at the 5th, 50th, and 95th percentiles, and the dose–response relationship between serum PLP concentrations and sleep duration. *p* < 0.05 (two-sided) was considered statistically significant using Stata 15.0.

## 3. Results

Table 1 lists the characteristics of each serum PLP concentrations quartile for participants. Except for the sampling season, significant differences were seen in the distribution of participants in other characteristics across quintiles of serum PLP concentrations. Compared with participants with lower serum PLP concentrations, participants in the higher PLP group were more likely to be male and married/cohabiting, had higher levels of education, and did higher intensity physical activity. In addition, participants in the lowest quartile of serum PLP concentrations were more likely to be poor, obese, have diabetes, and have depressive symptoms. Participants in the highest quartile of serum PLP concentrations were more likely to drink alcohol and less likely to smoke.

The relationships between serum PLP concentrations and sleep quality are shown in Table 2. In Model 2, the OR values between serum PLP concentrations and sleep disorders, trouble falling asleep, and waking up during the night were not statistically significant. However, compared with Q1, the ORs for daytime sleepiness of serum PLP concentrations in the Q2 and Q3 were 0.76 (0.59–0.99) and 0.78 (0.62–0.98), respectively. In the analysis stratified by sex, serum PLP concentrations were negatively associated with daytime sleepiness in males, with the OR (95% CI) of 0.65 (0.45–0.93) in Q4, whereas no significant association was found in females (Table 3). In different age groups, an inverse association of serum PLP concentration with daytime sleepiness was only observed in participants aged 40–59 years (OR = 0.62, 95% CI: 0.43–0.90) (Table S2). The results of other sleep quality-related problems in Model 2 are not statistically significant by either sex or age stratification (Tables S3 and S4), except for trouble falling asleep, which is negatively associated with serum PLP concentrations in older adults (≥60 years old) with the ORs of 0.68 (0.48–0.97) in Q2 and 0.69 (0.49–0.96) in Q3 (Table 4).

**Figure 2:**
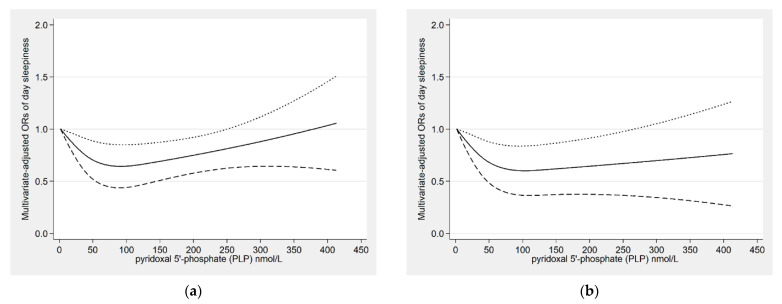
Restricted cubic spline model of the odds ratios (ORs) of day sleepiness with pyridoxal 5′-phosphate (PLP) concentration for the overall population (**a**) and males (**b**). The solid line and dashed lines represent the estimated ORs and the 95% confidence intervals.

There was a non-linear relationship between serum PLP concentrations and the risk of daytime sleepiness (*P*_for-nonlinearity_ = 0.019) (Figure 2a). We speculated that when the serum PLP concentration was within about 220 nmol/L, the negative correlation was statistically significant. To further explore the dose–response relationship in males, the result was prepared as Figure 2b (*P*_for-nonlinearity_ = 0.015). Within a certain range, for both males and females, PLP was negatively associated with daytime sleepiness, and we speculated that the OR value was the lowest when the serum PLP concentration was about 75 nmol/L.

The relationship between serum PLP concentrations and sleep duration is shown in Table 5. Compared with normal sleep duration (7–<9 h/night), serum PLP concentrations were negatively associated with the risks of very short sleep, short sleep, and long sleep duration. In Model 2, the relative risk ratios (RRRs) with the corresponding 95%CI were 0.58 (0.43–0.81) for very short sleep duration (Q4), 0.71 (0.61–0.83) for short sleep duration (Q4), and 0.62 (0.34–0.94) for long sleep duration (Q3). Table S5 lists the differences between the sexes. Serum PLP concentrations were only negatively associated with very short sleep (RRR = 0.66, 95% CI: 0.45–0.97) and short sleep duration (RRR = 0.68, 95% CI: 0.52–0.89) in males. In the age stratification analyses, serum PLP concentrations were related to very short and short sleep duration (0.41 CI: 0.21–0.79, 0.74 CI: 0.59–0.93) among participants older than 60 years old, and only negatively related to short sleep (0.61 CI: 0.46–0.81) among participants 18–39 years old (Table S6).

**Figure 3:**
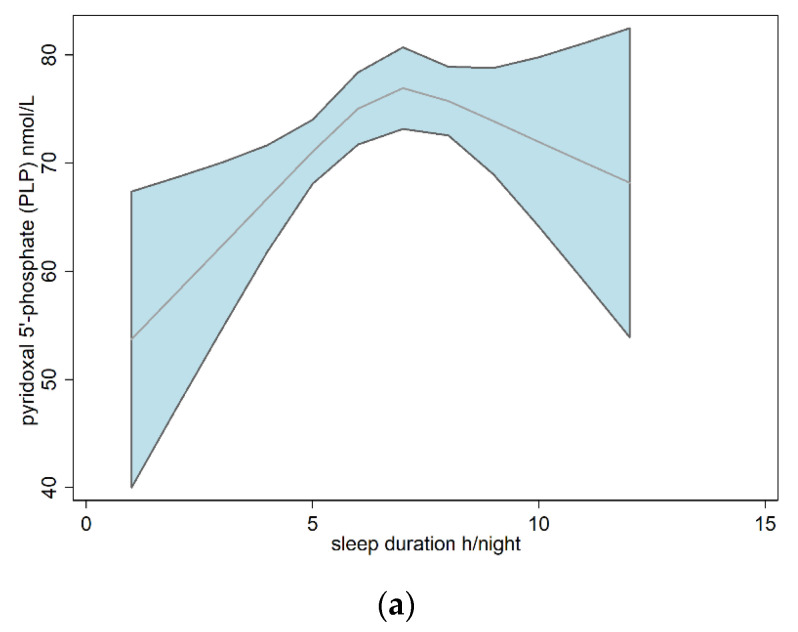

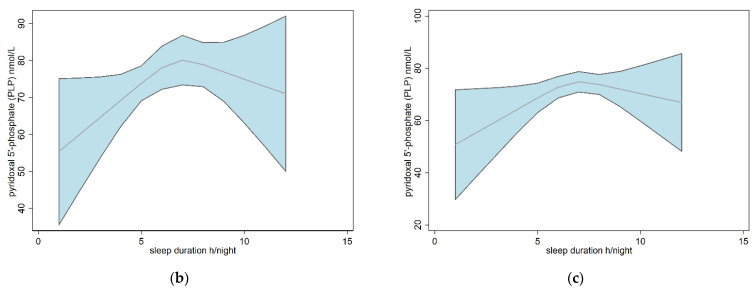
Dose–response relationships between sleep duration and pyridoxal 5′-phosphate (PLP) based on restricted cubic spline models for the overall population (**a**), males (**b**), and females (**c**). The red line and blue area represent the average concentration of PLP and the 95% confidence interval.

The dose–response relationship between serum PLP concentrations and sleep duration is shown in Figure 3a, presenting an inverted U-shape trend. We found similar relationships for both sexes (Figure 3b,c). Average serum PLP concentrations were highest in both males and females with normal sleep duration (7–<9 h).

## 4. Discussion

To the best of our knowledge, this was the first study to examine the relationships between serum PLP concentration and sleep-related problems (sleep quality and sleep duration) in the general population. Our study manifested that serum PLP concentration is nonlinearly and negatively associated with sleep quality problems, mainly daytime sleepiness. An interesting finding was that this relationship was significant only in males, but not in females. With age stratification, serum PLP concentrations were negatively related with daytime sleepiness only in middle-aged people, and with difficulty falling asleep in elderly adults. In terms of sleep duration, serum PLP concentration was negatively associated with very short sleep, short sleep, and long sleep duration risks. Moreover, participants with normal sleep duration had the highest average serum PLP concentrations. This negative relationship was found in all three sleep durations mentioned above in females, but only in very short and short sleep durations in males. Serum PLP concentration was negatively associated with very short sleep and short sleep risk in older adults (≥60 years) with age stratification, whereas this relationship was only significant for short sleep duration in younger adults (18–39 years).

A previous observational study has found that vitamin B6 intake was lower in people with poor sleep quality than in people with good sleep quality [39]. Moreover, vitamin B6 supplements with melatonin have been demonstrated to help treat insomnia in a prospective pilot study [40]. In addition, a randomized controlled trial suggested that the combination of vitamin B6 and γ-glutamate can improve sleep quality [52]. These studies indirectly support our findings. In contrast, one study found no significant difference in sleep quality in the vitamin B6 group compared to the placebo group [42]. The results of another intervention study also showed that the vitamin B6-containing complex did not significantly improve sleep compared with the control group [41]. Furthermore, a study suggested that taking pyridoxine had no effect on melatonin secretion in men [53]. The reason for the inconsistent results may be the different target populations and sample sizes of the studies. In addition, differences in how the studies measured sleep performance, such as sleep quality, using a simple question or different questionnaires, also contributed to the inconsistency.

There are several potential mechanisms for the link between PLP and sleep. Firstly, PLP is a coenzyme that can participate in the synthesis of melatonin, which is generally considered an important hormone that affects sleep [54,55]. Specifically, PLP participates in the hydroxylation of tryptophan to generate 5-hydroxytryptophan (5-HTP) and decarboxylation to produce 5-HT, which is considered as a precursor for melatonin [56]. Secondly, PLP can affect the nervous system by participating in the synthesis of neurotransmitters [57], such as GABA, which may be involved in the regulation of sleep [58]. In addition, the anti-inflammatory and antioxidant effects of PLP are also possible mechanisms involved in sleep. Inflammation has been shown in studies to affect sleep [59,60], and some markers of inflammation also have a circadian rhythm [61]. During inflammation, PLP is mobilized to active inflammatory sites and regulates PLP-dependent enzymes and metabolic pathways, which play a role in the inflammatory response [62,63]. Furthermore, oxidative stress can disrupt sleep homeostasis through different mechanisms [64]. Vitamin B6, or PLP, is an antioxidant that protects against oxidative stress by directly scavenging free radicals or indirectly participating in the glutathione-dependent antioxidant system [34,57,65,66,67]. Furthermore, we observed sex and age differences in the association between serum PLP concentrations and the risk of sleep-related problems. The reason may be that there are differences in sleep rhythms and hormone levels among people of different genders and ages, which can affect sleep [51,68,69,70].

There are several advantages to this study. Firstly, this study used data from NHANES, a nationally representative database in the United States, and rigorous quality control was performed to ensure the authenticity and generalization of the results. Secondly, we used serum PLP, a biomarker of vitamin B6, which reflects biological effective exposure. Thirdly, we explored potential dose–response relationships between serum PLP and sleep-related problems. However, there are some potential limitations. The first is the disadvantage of the cross-sectional design, which cannot infer causality. The exact protective effect of B6 on sleep-related problems requires further prospective studies to determine. Secondly, sleep-related problems were self-reported, which may not reflect objective sleep conditions and lead to information bias. Finally, some residual confounding may influence the results, although as many influencing factors as possible were included in this study, including demographic characteristics, lifestyle, dietary factors, and some diseases.

## 5. Conclusions

Our study suggested that serum PLP concentrations were nonlinearly and negatively associated with the risk of daytime sleepiness, especially in males. In addition, serum PLP concentrations were negatively related to very short, short, and long sleep durations.

## Figures and Tables

**Table 1:** Baseline characteristics of participants by quartile of pyridoxal 5′-phosphate (PLP) (NHANES 2005–2008).

Characteristics	Quartiles Plasma Pyridoxal 5′-Phosphate (PLP) (nmol/L)				*p*-Value
	Q1 (<26.4)	Q2 (26.4–43.4)	Q3 (43.4–74.8)	Q4 (≥74.8)	
	*n* = 3804	*n* = 3815	*n* = 3790	*n* = 3797	
Gender (%) ^a^					
Male	1533 (37.63)	1891 (48.66)	2209 (57.24)	2120 (53.65)	<0.001
Female	2271 (62.37)	1924 (51.33)	1581 (42.76)	1677 (46.35)	
Age (%) ^a^					
18–39 years	1125 (31.83)	1579 (42.66)	1630 (43.01)	1351 (37.15)	<0.001
40–59 years	1222 (40.39)	1212 (38.60)	1157 (37.70)	1103 (36.62)	
≥60 year	1457 (27.76)	1024 (18.74)	1003 (19.28)	1343 (26.23)	
Race/ethnicity (%) ^a^					
Mexican American	610 (7.22)	828 (10.00)	836 (9.87)	598 (6.43)	
Other Hispanic	286 (3.83)	372 (5.45)	349 (4.98)	294 (3.91)	
Non-Hispanic White	1724 (67.54)	1610 (65.17)	1723 (69.11)	2115 (76.13)	<0.001
Non-Hispanic Black	1058 (16.83)	817 (12.33)	683 (9.53)	602 (7.45)	
Other races	126 (4.58)	188 (7.05)	199 (6.51)	188 (6.08)	
Educational level (%) ^a^					
<high school	1379 (25.26)	1254 (22.43)	1075 (18.57)	814 (13.05)	<0.001
High school	1031 (29.87)	932 (24.94)	914 (24.18)	816 (20.76)	
>high school	1386 (44.87)	1627 (52.63)	1796 (57.26)	2163 (66.19)	
Ratio of income to poverty (%) ^a^					
<1	959 (18.21)	808 (14.15)	695 (11.92)	503 (7.86)	<0.001
≥1	2845 (81.79)	3007 (85.84)	3095 (88.08)	3294 (92.14)	
Marital status (%) ^a^					
Married/Cohabiting	1983 (58.91)	2158 (63.91	2218 (66.19)	2263 (66.23)	<0.001
Windowed/Living alone	1741 (41.09)	1514 (36.09)	1394 (33.81)	1410 (33.77)	
Body mass index (%) ^a^					
<25 kg/m^2^	971 (26.81)	1098 (30.02)	1204 (32.93)	1371 (38.88)	<0.001
25 to <30 kg/m^2^	1026 (25.37)	1218 (31.40)	1372 (35.88)	1430 (37.37)	
≥30 kg/m^2^	1807 (47.81)	1499 (38.58)	1214 (31.20)	996 (23.75)	
Physical activity (%) ^a^					
Vigorous	882 (26.80)	1284 (37.29)	1536 (44.33)	1588 (45.78)	<0.001
Moderate	1142 (32.22)	1080 (29.83)	1086 (30.72)	1197 (32.61)	
Other	1780 (40.98)	1450 (32.88)	1168 (24.95)	1012 (21.61)	
Depressive symptoms (%) ^a^	397 (10.95)	279 (6.68)	201 (4.85)	179 (3.50)	<0.001
Diabetes (%) ^a^	861 (18.52)	616 (11.78)	505 (10.20)	492 (9.21)	<0.001
Hypertension (%) ^a^	2194 (53.96)	1786 (44.11)	1726 (44.24)	1847 (45.17)	<0.001
Caffeine intake (mg/d) ^b^	103 (212)	94 (172)	86 (152)	96.5 (173)	<0.001
Total energy (kcal/day) ^b^	1772.5 (180.5)	1891.5 (166)	2591 (164)	1977 (188)	<0.001
Smoke at least 100 cigarettes in life (%) ^a^	2020 (56.11)	1677 (48.63)	1513 (44.21)	1485 (40.02)	<0.001
Had at least 12 alcohol drinks a year (%) ^a^	2133 (68.03)	2261 (75.85)	2387 (78.23)	2539 (79.51)	<0.001
Sampling season (%) ^a^					
November to April	1825 (42.90)	1824 (40.04)	1697 (39.29)	1595 (37.30)	0.081
May to October	1979 (57.10)	1991 (59.96)	2093 (60.71)	2202 (62.70)	

Data are represented the number of subjects (weighted percentage) or median (interquartile range). ^a^ Chi-square test was used to compare the percentages between participants in different quartiles of serum PLP concentrations. ^b^ Kruskal–Wallis test were used to compare the medians between participants in different quartiles of serum PLP concentrations.

**Table 2:** Weighted odds ratios (95% confidence intervals) for sleep disorders across quartiles of pyridoxal 5′-phosphate (PLP) concentrations (NHANES 2005–2008).

	Cases/ Participants	Crude	Model 1 ^a^	Model 2 ^b^
Sleep disorders				
Q1 (<27.3)	195/2428	1.00 (ref)	1.00 (ref)	1.00 (ref)
Q2 (27.3 to <44.0)	153/2430	0.74 (0.54–1.03)	0.77 (0.55–1.07)	0.78 (0.53–1.16)
Q3 (44.0 to <76.3)	141/2425	0.67 (0.49–0.90) **	0.67 (0.49–0.93) **	0.88 (0.61–1.28)
Q4 (≥76.3)	150/2427	0.75 (0.55–1.03)	0.74 (0.53–1.03)	1.02 (0.66–1.58)
Trouble falling asleep				
Q1 (<27.3)	447/2428	1.00 (ref)	1.00 (ref)	1.00 (ref)
Q2 (27.3 to <44.0)	385/2430	0.77 (0.66–0.90) **	0.81 (0.69–0.96) *	0.93 (0.76–1.14)
Q3 (44.0 to <76.3)	334/2425	0.66 (0.54–0.79) **	0.72 (0.58–0.89) **	0.93 (0.72–1.19)
Q4 (≥76.3)	342/2427	0.70 (0.58–0.84) **	0.76 (0.63–0.93) **	1.09 (0.87–1.36)
Wake up during the night				
Q1 (<27.3)	541/2428	1.00 (ref)	1.00 (ref)	1.00 (ref)
Q2 (27.3 to <44.0)	429/2430	0.72 (0.57–0.90) **	0.79 (0.63–0.98) **	0.92 (0.69–1.22)
Q3 (44.0 to <76.3)	424/2425	0.68 (0.59–0.80) **	0.77 (0.66–0.90) **	0.98 (0.79–1.22)
Q4 (≥76.3)	404/2427	0.63 (0.53–0.75) **	0.69 (0.58–0.82) **	0.89 (0.73–1.10)
Daytime sleepiness				
Q1 (<27.3)	503/2428	1.00 (ref)	1.00 (ref)	1.00 (ref)
Q2 (27.3 to <44.0)	382/2430	0.66 (0.55–0.79) **	0.67 (0.55–0.80) **	0.76 (0.59–0.99) *
Q3 (44.0 to <76.3)	368/2425	0.62 (0.51–0.75) **	0.64 (0.53–0.77) **	0.78 (0.62–0.98) *
Q4 (≥76.3)	373/2427	0.59 (0.51–0.69) **	0.63 (0.54–0.72) **	0.80 (0.64–1.00)

Calculated using binary logistic regression models. ^a^ Model 1 adjusted for age and sex. ^b^ Model 2 adjusted for age, sex, race/ethnicity, education level, household poverty ratio, marital status, body mass index, physical activity, smoking status, caffeine intake, energy, alcohol consumption, hypertension, diabetes, depressive symptoms, and sampling season. * *p* < 0.05; ** *p* < 0.01.

**Table 3:** Weighted odds ratios (95% confidence intervals) for daytime sleepiness across quartiles of pyridoxal 5′-phosphate (PLP) concentrations stratified by gender (NHANES 2005–2008).

	Model 2 ^a^	
	Males	Females
Q1 (<27.3)	1.00 (ref)	1.00 (ref)
Q2 (27.3 to <44.0)	0.68 (0.46–1.02)	0.81 (0.58–1.13)
Q3 (44.0 to <76.3)	0.72 (0.53–0.97) *	0.82 (0.58–1.16)
Q4 (≥76.3)	0.65 (0.45–0.93) *	0.95 (0.70–1.30)

Calculated using binary logistic regression models. ^a^ Model 2 adjusted for age, sex, race/ethnicity, education level, household poverty ratio, marital status, body mass index, physical activity, smoking status, caffeine intake, energy, alcohol consumption, hypertension, diabetes, depressive symptoms, and sampling season. * *p* < 0.05.

**Table 4:** Weighted odds ratios (95% confidence intervals) for having trouble falling asleep across quartiles of pyridoxal 5′-phosphate (PLP) concentrations stratified by age (NHANES 2005–2008).

	Model 2 ^a^		
	18 ≤ Age < 40 Years	40 ≤ Age < 60 Years	Age ≥ 60 Years
Q1 (<27.3)	1.00 (ref)	1.00 (ref)	1.00 (ref)
Q2 (27.3 to <44.0)	1.03 (0.64–1.64)	0.93 (0.62–1.40)	0.68 (0.48–0.97) *
Q3 (44.0 to <76.3)	0.95 (0.66–1.37)	1.01 (0.69–1.49)	0.69 (0.49–0.96) *
Q4 (≥76.3)	0.82 (0.52–1.30)	1.56 (0.97–2.50)	0.75 (0.51–1.10)

Calculated using binary logistic regression models. ^a^ Model 2 adjusted for age, sex, race/ethnicity, education level, household poverty ratio, marital status, body mass index, physical activity, smoking status, caffeine intake, energy, alcohol consumption, hypertension, diabetes, depressive symptoms, and sampling season. * *p* < 0.05.

**Table 5:** Weighted relative risk ratios (95% CIs) for sleep duration (reference, 7–<9 h/night) across quartiles of pyridoxal 5′-phosphate (PLP) concentrations (NHANES 2005–2010).

Pyridoxal 5′-Phosphate (PLP) (nmol/L)	Model 2 ^a^		
	Very Short Sleep (<5 h/Night)	Short Sleep (5–<7 h/Night)	Long Sleep (≥9 h/Night)
Q1 (<26.4)	1.00 (ref)	1.00 (ref)	1.00 (ref)
Q2 (26.4 to <43.4)	0.73 (0.54–1.00)	0.79 (0.68–0.92) **	0.83 (0.53–1.30)
Q3 (43.4 to <74.8)	0.58 (0.45–0.76) **	0.74 (0.65–0.85) **	0.62 (0.34–0.94) *
Q4 (≥74.8)	0.58 (0.43–0.81) **	0.71 (0.61–0.83) **	0.67 (0.40–1.02)

Calculated using multinomial logistic regression models. ^a^ Model 2 adjusted for age, sex, race/ethnicity, education level, household poverty ratio, marital status, body mass index, physical activity, smoking status, caffeine intake, energy, alcohol consumption, hypertension, diabetes, depressive symptoms, and sampling season. * *p* < 0.05; ** *p* < 0.01.

## Data Availability

The datasets supporting the conclusions of this article are publicly available from the NHANES (https://www.cdc.gov/nchs/nhanes/index.htm (accessed on 19 April 2022)).

## Supplementary Materials

The following supporting information can be downloaded at: https://www.mdpi.com/article/10.3390/nu14173516/s1. Table S1: The classifications of categorical covariates. Table S2: Weighted odds ratios (95% confidence intervals) for day sleepiness across quartiles of pyridoxal 5′-phosphate (PLP) concentrations stratified by age (NHANES 2005–2008). Table S3: Weighted odds ratios (95% confidence intervals) for other sleep quality problems across quartiles of pyridoxal 5′-phosphate (PLP) concentrations stratified by gender (NHANES 2005–2008). Table S4: Weighted odds ratios (95% confidence intervals) for other sleep quality problems across quartiles of pyridoxal 5′-phosphate (PLP) concentrations stratified by age (NHANES 2005–2008). Table S5: Weighted relative risk ratios (95% confidence intervals) for sleep duration (reference, 7 ≤ 9 h/night) across quartiles of pyridoxal 5′-phosphate (PLP) concentrations stratified by gender (NHANES 2005–2010). Table S6: Weighted relative risk ratios (95% confidence intervals) for sleep duration (reference, 7 ≤ 9 h/night) across quartiles of pyridoxal 5′-phosphate (PLP) concentrations stratified by age (NHANES 2005–2010).
