# Supplementary material for: Association of Pyridoxal 5′-Phosphate with Sleep-Related Problems in a General Population
Source: Nutrients. 2022 Aug 26;14(17):3516. doi: 10.3390/nu14173516 (PMC9460331; doi:10.3390/nu14173516)
Supplement: Supplementary file 1 [file nutrients-14-03516-s001.zip › nutrients-1853776-supplementary.pdf]

Table S1. The classifications of categorical covariates.

Covariates	Classifications				
Gender	Males		Females		
Age groups	18–39 years		40–59 years	≥60years	
Race/ethnicity	Mexican American	Other Hispanic	Non-Hispanic White	Non-Hispanic Black	Other races
Educational level	Below high school		High school	Above high school	
Ratio of income to poverty	<1		≥1		
Marital status	Married/Living with partner		Windowed/Divorced/Separated/ Never married		
Body mass index	< 25 kg/m2		25 to <30 kg/m2	≥30 kg/m2	
Physical activity	Vigorous		Moderate	Other	
Depressive symptoms	Yes		No		
Diabetes	Yes		No		
Hypertension	Yes		No		
Caffeine intake	Continuous variable				
Total energy	Continuous variable				
Smoke at least 100 cigarettes in life	Yes		No		
Had at least 12 alcohol drink a year	Yes		No		
Examination time	November 1 through April 30		May 1 through October 31		

Table S2. Weighted odds ratios (95% confidence intervals) for day sleepiness across quartiles of pyridoxal 5'-phosphate (PLP) concentrations stratified by age (NHANES 2005–2008).

	**Model 2 [a]**		
	**18 ≤ Age < 40 Years**	**40 ≤ Age < 60Years**	**Age ≥ 60Years**
Q1 (<27.3)	1.00 (ref)	1.00 (ref)	1.00 (ref)
Q2 (27.3 to <44.0)	0.76 (0.49–1.18)	0.82 (0.55–1.23)	0.67 (0.44–1.01)
Q3 (44.0 to <76.3)	0.90 (0.65–1.26)	0.62 (0.43–0.90) *	0.93 (0.66–1.32)
Q4 (≥76.3)	0.71 (0.48–1.06)	0.87 (0.57–1.33)	0.92 (0.64–1.33)

Calculated using binary logistic regression models. [a] Model 2 adjusted for age, sex, race/ethnicity, education level, household poverty ratio, marital status, body mass index, physical activity, smoking status, caffeine intake, energy, alcohol consumption, hypertension, diabetes, depressive symptoms, and sampling season. * $p < 0.05$.

Table S3. Weighted odds ratios (95% confidence intervals) for other sleep quality problems across quartiles of pyridoxal 5'-phosphate (PLP) concentrations stratified by gender (NHANES 2005–2008).

	Model 2 [a]	
	**Males**	**Females**
**Sleep disorders**		
Q1 (<27.3)	1.00 (ref)	1.00 (ref)
Q2 (27.3 to <44.0)	0.64 (0.39–1.04)	0.98 (0.58–1.66)
Q3 (44.0 to <76.3)	0.77 (0.48–1.24)	1.01 (0.59–1.74)
Q4 (≥76.3)	0.88 (0.48–1.59)	1.23 (0.76–2.01)
**Trouble falling asleep**		
Q1 (<27.3)	1.00 (ref)	1.00 (ref)
Q2 (27.3 to <44.0)	0.95 (0.68–1.33)	0.90 (0.67–1.20)
Q3 (44.0 to <76.3)	0.74 (0.52–1.07)	1.08 (0.79–1.48)
Q4 (≥76.3)	1.00 (0.75–1.35)	1.13 (0.85–1.50)
**Wake up during night**		
Q1 (<27.3)	1.00 (ref)	1.00 (ref)
Q2 (27.3 to <44.0)	0.90 (0.58–1.40)	0.90 (0.69–1.17)
Q3 (44.0 to <76.3)	0.84 (0.58–1.22)	1.06 (0.84–1.36)
Q4 (≥76.3)	0.78 (0.57–1.07)	0.96 (0.73–1.27)

Calculated using binary logistic regression models. [a] Model 2 adjusted for age, sex, race/ethnicity, education level, household poverty ratio, marital status, body mass index, physical activity, smoking status, caffeine intake, energy, alcohol consumption, hypertension, diabetes, depressive symptoms, and sampling season.

Table S4. Table S3. Weighted odds ratios (95% confidence intervals) for other sleep quality problems across quartiles of pyridoxal 5'-phosphate (PLP) concentrations stratified by age (NHANES 2005–2008).

	Model 2 [a]		
	**18 ≤ Age < 40 Years**	**40 ≤ Age < 60Years**	**Age ≥ 60Years**
**Sleep disorder**			
Q1 (<27.3)	1.00 (ref)	1.00 (ref)	1.00 (ref)
Q2 (27.3 to <44.0)	0.57 (0.27–1.22)	0.77 (0.41–1.45)	0.86 (0.55–1.36)
Q3 (44.0 to <76.3)	0.76 (0.33–1.75)	1.01 (0.53–1.92)	0.69 (0.46–1.05)
Q4 (≥76.3)	0.87 (0.32–2.39)	1.18 (0.54–2.57)	0.84 (0.49–1.45)
**Wake up during night**			
Q1 (<27.3)	1.00 (ref)	1.00 (ref)	1.00 (ref)
Q2 (27.3 to <44.0)	0.84 (0.52–1.37)	0.86 (0.54–1.39)	1.02 (0.73–1.42)
Q3 (44.0 to <76.3)	0.81 (0.51–1.30)	1.02 (0.75–1.39)	1.05 (0.80–1.38)
Q4 (≥76.3)	0.75 (0.48–1.16)	0.95 (061–1.48)	0.97 (0.68–1.39)

Calculated using binary logistic regression models. [a] Model 2 adjusted for age, sex, race/ethnicity, education level, household poverty ratio, marital status, body mass index, physical activity, smoking status, caffeine intake, energy, alcohol consumption, hypertension, diabetes, depressive symptoms, and sampling season.

Table S5. Weighted relative risk ratios (95% confidence intervals) for sleep duration (reference, 7–<9h/night) across quartiles of pyridoxal 5'-phosphate (PLP) concentrations stratified by gender (NHANES 2005–2010).

Pyridoxal 5'-Phosphate (PLP) (nmol/L)	Model 2 [a]		
	**Very Short Sleep (<5 h/Night)**	**Short Sleep (5–<7 h/Night)**	**Long Sleep (≥9 h/Night)**
**Males**			
Q1 (<26.4)	1.00 (ref)	1.00 (ref)	1.00 (ref)
Q2 (26.4to <43.4)	0.86 (0.57–1.30)	0.79 (0.60–1.03)	0.84 (0.47–1.48)
Q3 (43.4to <74.8)	0.66 (0.45–0.97) *	0.75 (0.60–0.93) **	0.61 (0.30–1.23)
Q4 (≥74.8)	0.65 (0.41–1.04)	0.68 (0.52–0.89) **	0.63 (0.35–1.14)
**Females**			
Q1 (<26.4)	1.00 (ref)	1.00 (ref)	1.00 (ref)
Q2 (26.4to <43.4)	0.62 (0.42–0.91) *	0.79 (0.69–0.91) **	0.83 (0.46–1.50)
Q3 (43.4to <74.8)	0.51 (0.34–0.77) **	0.72 (0.58–0.88) **	0.53 (0.29–0.98) *
Q4 (≥74.8)	0.52 (0.29–0.95) *	0.73 (0.59–0.90) **	0.66 (0.33–1.33)

Calculated using multinomial logistic regression models. [a] Model 2 adjusted for age, sex, race/ethnicity, education level, household poverty ratio, marital status, body mass index, physical activity, smoking status, caffeine intake, energy, alcohol consumption, hypertension, diabetes, depressive symptoms, and sampling season. * $p < 0.05$; ** $p < 0.01$.

Table S6. Weighted relative risk ratios (95% confidence intervals) for sleep duration (reference, 7–<9h/night) across quartiles of pyridoxal 5'-phosphate (PLP) concentrations stratified by age (NHANES 2005–2010).

Pyridoxal 5'-Phosphate (PLP) (nmol/L)	Model 2 [a]		
	**Very Short Sleep (<5 h/Night)**	**Short Sleep (5–<7 h/Night)**	**Long Sleep (≥9 h/Night)**
18 ≤ Age < 40 Years			
Q1 (<26.4)	1.00 (ref)	1.00 (ref)	1.00 (ref)
Q2 (26.4to <43.4)	0.76 (0.51–1.14)	0.77 (0.64–0.92) **	1.46 (0.72–2.92)
Q3 (43.4to <74.8)	0.62 (0.34–1.12)	0.64 (0.48–0.85) **	0.55 (0.26–1.15)
Q4 (≥74.8)	0.87 (0.47–1.60)	0.61 (0.46–0.81) **	0.64 (0.29–1.45)
40 ≤ Age < 60 Years			
Q1 (<26.4)	1.00 (ref)	1.00 (ref)	1.00 (ref)
Q2 (26.4to <43.4)	0.81 (0.50–1.32)	0.82 (0.63–1.06)	0.28 (0.10–0.76) *
Q3 (43.4to <74.8)	0.64 (0.40–1.02)	0.83 (0.68–1.00) *	0.32 (0.10–1.04)
Q4 (≥74.8)	0.49 (0.30–0.79) **	0.71 (0.54–0.93) *	0.48 (0.22–1.08)
Age ≥ 60Years			
Q1 (<26.4)	1.00 (ref)	1.00 (ref)	1.00 (ref)
Q2 (26.4to <43.4)	0.53 (0.25–1.11)	0.71 (0.54–0.94) *	0.81 (0.48–1.37)
Q3 (43.4to <74.8)	0.53 (0.29–0.96) *	0.74 (0.59–0.93) *	0.95 (0.45–1.98)
Q4 (≥74.8)	0.41 (0.21–0.79) **	0.79 (0.62–1.02)	0.78 (0.37–1.63)

Calculated using multinomial logistic regression models. [a] Model 2 adjusted for age, sex, race/ethnicity, education level, household poverty ratio, marital status, body mass index, physical activity, smoking status, caffeine intake, energy, alcohol consumption, hypertension, diabetes, depressive symptoms, and sampling season. * $p < 0.05$; ** $p < 0.01$.
